# Clinical predictors of postpartum vaginal and vulvar hematomas and the need for surgical treatment

**DOI:** 10.1002/pmf2.70316

**Published:** 2026-06-05

**Authors:** Itai Atar, Einat Tako, Daniel Gabbai, Itamar Gilboa, Yariv Yogev, Emmanuel Attali

**Affiliations:** ^1^ Lis Maternity Hospital Tel Aviv Sourasky Medical Center affiliated to the Gray Faculty of Medicine and Health Sciences Tel Aviv University Tel Aviv Israel

**Keywords:** episiotomy, perineal trauma, postpartum complication, postpartum hematoma, vacuum delivery

## Abstract

**Objective:**

To identify risk factors for postpartum vaginal or vulvar hematoma and to determine clinical predictors of surgical intervention.

**Study design:**

A retrospective matched case‐control study was conducted at a tertiary, university‐affiliated center (2012–2024). Women diagnosed with vaginal or vulvar hematoma (cases) during the postpartum hospitalization were matched in a 1:3 ratio by maternal and gestational age to controls. Independent risk factors were identified using conditional logistic regression with a Cox regression model. A risk score was derived from the multivariable model, and ROC analysis was used to assess performance. A subgroup analysis evaluated predictors of surgery.

**Results:**

Among 147,045 deliveries, 57 women (0.04%) had postpartum hematoma; 170 matched controls were included. Mean diagnosis time was 1.0 ± 1.6 days post‐partum. Hematoma was associated with vacuum‐assisted delivery (21.1% vs. 4.7%, *p* < 0.001), episiotomy (24.6% vs. 2.4%, *p* < 0.001), second‐degree laceration (38.6% vs. 15.3%, *p* < 0.001), nulliparity, and lower body mass index (BMI; *p* < 0.001). In multivariable analysis, episiotomy (odds ratio [OR] 35.9, 95% confidence interval [CI] 5.6–232.4), second‐degree laceration (OR 10.3, 95% CI 3.2–32.9), and vacuum delivery (OR 5.0, 95% CI 1.0–24.5) independently increased risk, whereas higher BMI (OR 0.69/unit, 95% CI 0.55–0.85) and epidural analgesia (OR 0.33, 95% CI 0.12–0.93) were protective. The model's AUC was 0.83; a score ≥3 yielded 68% sensitivity and 79% specificity and a 97.8% negative predictive value, supporting its utility in ruling out hematoma and reducing surgical interventions. Among women with a hematoma, 26.3% required surgery. Mean time to surgery from diagnosis averaged 6.5 ± 3.8 h. Prolonged second stage (20% vs. 2.4%, *p* < 0.05) was significantly associated with surgery, with trends toward more vacuum deliveries, no epidural, and greater hemoglobin decline. Postpartum hemorrhage and blood transfusion rates did not differ between groups.

**Conclusion:**

Postpartum hematoma is rare but associated with vacuum delivery, episiotomy, second‐degree laceration, and low BMI, while epidural analgesia is protective. Early recognition supports timely management.

## 1 INTRODUCTION

Postpartum vaginal and vulvar hematomas represent a potentially serious but under‐recognized complication of vaginal delivery, with significant implications for maternal morbidity and the need for surgical intervention [1]. These collections of blood within the vaginal, vulvar, or paravaginal tissues can lead to severe pain, hemodynamic instability, and, in some cases, life‐threatening hemorrhage requiring emergency management [2, 3]. Although their clinical significance, postpartum hematomas remain relatively uncommon, with reported incidence rates varying from 0.05% to 0.1% of vaginal deliveries in large population‐based studies [4, 5].

The management of postpartum hematomas has evolved from primarily surgical approaches to more nuanced, individualized strategies that consider hematoma location, size, and patient stability. Small, stable hematomas in hemodynamically stable patients may be managed conservatively with close monitoring, while expanding or large hematomas typically require intervention [6]. A significant advancement in management has been the introduction of selective arterial embolization as an essential treatment option, particularly valuable for upper vaginal and paravaginal hematomas [7].

Current understanding of risk factors for postpartum hematomas derives from a combination of case series and observational studies, though large‐scale analyses remain limited. Literature consistently identifies associations with operative vaginal delivery, episiotomy, prolonged second stage of labor, fetal macrosomia, and primiparity, but the relative importance of these factors and their interactions have not been well characterized in adequately powered studies [4, 8, 9]. Given the limitations of existing literature and the clinical importance of this complication, there is a clear need for comprehensive studies that can provide robust evidence for risk factor identification and clinical decision‐making [3, 4].

This study aims to address these knowledge gaps by conducting a large‐scale matched case–control analysis to identify independent risk factors for postpartum hematomas and develop a useful risk prediction model. Additionally, we aimed to identify factors associated with the need for surgical intervention among women who develop postpartum hematomas to facilitate a more accurate risk assessment and guide clinical management.

## 2 MATERIALS AND METHODS

We conducted a retrospective matched case‐control study at a single University‐affiliated tertiary academic medical center between January 2012 and December 2024.

The study population was composed of pregnant patients who were diagnosed with vaginal or vulvar hematoma during postpartum hospitalization, which were identified through medical record review using ICD‐10 codes and manual chart verification. Diagnosis was based on clinical findings, which were consistent with vaginal or vulvar hematoma documented by physicians, such as palpable masses or localized swelling, and when performed, confirmed by imaging, mainly ultrasound. For each case, three controls were selected from women who delivered during the same period, matched by maternal age and gestational age, with no documented postpartum hematoma.

Initial data extraction was performed via an automated retrieval process from computerized delivery room logbooks, providing a comprehensive set of data, including maternal demographics (age, body mass index, parity, and medical comorbidities); pregnancy characteristics (gestational age, estimated fetal weight, and pregnancy complications); labor and delivery factors (induction, epidural use, duration of labor stages, and mode of delivery); interventions (episiotomy and instrumental delivery by vacuum); perineal trauma (classified as first‐, second‐, and obstetric anal sphincter injury OASI]); and postpartum outcomes, including hemoglobin change, blood transfusion, and length of hospital stay. To ensure accuracy, all extracted variables were manually verified by the research team during the data coding phase prior to statistical analysis.

Management decisions were categorized as conservative (observation and analgesics) or surgical (evacuation, drainage, or arterial embolization) based on clinical (pain severity and hemodynamic status) and laboratory parameters (hemoglobin decline).

The primary outcome was the diagnosis of postpartum hematoma during hospitalization, defined as a collection of blood within the vaginal, vulvar, or paravaginal tissues confirmed by clinical examination and/or imaging. The secondary outcome was the need for surgical intervention among women with hematoma, including surgical evacuation, drainage, or transcatheter arterial embolization. Exposure variables included maternal demographics and obstetric characteristics, pregnancy complications, and intrapartum factors. These included maternal age, BMI, parity, smoking status, gestational age, newborn birth weight, and the presence of gestational diabetes (GDM), intrauterine growth restriction (IUGR), or preeclampsia. Intrapartum variables included induction or augmentation with oxytocin, epidural anesthesia, duration of second stage, instrumental delivery, episiotomy, and degree of perineal laceration. Additional interventions such as manual removal of placenta, uterine revision, and postpartum blood transfusion were also recorded. Low‐dose aspirin use was documented in only three patients (*n* = 1 in the hematoma group, *n* = 2 in the control group) and was therefore excluded from further statistical analysis.

### 2.1 Statistical analysis

Patients' characteristics were summarized with descriptive statistics. Normally distributed quantitative variables were described using mean (± standard deviation [SD]) and non‐normally distributed variables using median (interquartile range [IQR]). Histograms, *Q*–*Q* plots, and Kolmogorov–Smirnov tests determined distribution normality. Univariate analysis techniques identified group differences.

Univariate analyses for both dichotomous and continuous variables were conducted using conditional logistic regression via Cox regression. This approach was chosen to preserve the matched case–control design.

Multivariable conditional logistic regression was performed to identify independent risk factors, with variables selected based on clinical relevance and statistical significance in univariate analysis (*p* < 0.05). A risk prediction score for developing postpartum hematoma was derived from the final multivariable model, and receiver operating characteristic (ROC) analysis was performed to assess discriminative ability. Because the case–control design does not preserve disease prevalence, positive and negative predictive values (PPV and NPV) were not calculated directly from the sampled dataset. Instead, PPV and NPV were derived using the model's sensitivity and specificity together with the observed population prevalence of postpartum hematoma at our institution, applying standard Bayesian predictive value formulas to reflect real‐world clinical performance.

Missing data were handled using complete–case analysis. Because the proportion of missing values was low, no imputation procedures were applied, and the risk of bias due to missingness was considered minimal.

All analyses were performed using IBM SPSS Statistics for Windows, Version 29.0 (IBM Corp.), with statistical significance set at *p* < 0.05.

The study was approved by the Institutional Review Board (IRBTLV‐0284‐08 July 31,2024), and informed consent was waived due to the retrospective nature of the analysis.

## 3 RESULTS

During the study period, 147,045 deliveries occurred at our institution, including 113,520 vaginal births. Among these, 57 women (0.039%) were diagnosed with postpartum hematomas and met inclusion criteria, alongside 170 matched controls. The mean maternal age was similar between groups (34.5 ± 4.6 vs. 34.6 ± 3.9 years), as was the mean gestational age at delivery (39.1 ± 1.8 weeks for both).

Women who developed postpartum hematomas differed significantly from controls in several baseline and delivery characteristics (Table 1). Cases had a significantly lower BMI (20.0 ± 5.0 vs. 23.2 ± 2.4, *p* < 0.001) and were more frequently nulliparous (71.9% vs. 46.5%, *p* < 0.001). Vacuum‐assisted delivery (21.1% vs. 4.7%, *p* < 0.001), episiotomy (24.6% vs. 2.4%, *p* < 0.001), and second‐degree perineal laceration (38.6% vs. 15.3%, *p* < 0.001) were significantly more common among cases, as was a prolonged second stage of labor (5.3% vs. 0.6%, *p* < 0.05). Women diagnosed with hematoma were more likely to deliver without epidural analgesia (54.4% vs. 71.8%, *p* < 0.05), and blood transfusion was required more often among cases (15.8% vs. 2.4%, *p* = 0.02).

**TABLE 1:** Baseline and intrapartum characteristics of women with and without postpartum hematoma.

Characteristic	Postpartum hematoma, *N* = 57	Control group (no hematoma), *N* = 170	*p*‐value
Maternal age, years (mean, SD)	34.5 (4.6)	34.6 (3.9)	–
Gestational age, weeks (mean, SD)	39.1 (1.8)	39.1 (1.9)	–
Newborn weight, g (mean, SD)	3162 (491)	3245 (462)	0.19
BMI (mean, SD)	20 (5.03)	23.21 (2.43)	**<0.001**
Nulliparity (*n*, %)	41 (71.9)	79 (46.5)	**<0.001**
Smoking (*n*, %)	1 (1.8)	9 (5.3)	0.30
Previous CD (*n*, %)	4 (7.0)	7 (4.1)	0.34
Multiple gestation (*n*, %)	1 (1.8)	0 (0)	0.62
IUGR (*n*, %)	2 (3.5)	2 (1.8)	0.40
GDM (*n*, %)	6 (10.5)	16 (9.4)	0.83
PET (*n*, %)	2 (3.5)	7 (4.1)	0.80
Induction of labor (*n*, %)	13 (22.8)	25 (14.7)	0.20
Oxytocin use (*n*, %)	27 (47.4)	73 (42.9)	0.55
Epidural during delivery (*n*, %)	30 (52.6)	123 (72.4)	**0.008**
PSS (*n*, %)	4 (7)	6 (3.5)	0.27
Intrapartum fever (*n*, %)	2 (3.5)	4 (2.4)	0.62
Vacuum‐assisted delivery (*n*, %)	12 (21.1)	8 (4.7)	**<0.001**
Manual removal of placenta (*n*, %)	–	3 (1.8)	–
Revision of the uterus (*n*, %)	2 (3.5)	8 (4.7)	0.67
First degree laceration (*n*, %)	10 (17.5)	32 (18.8)	0.85
Second degree laceration (*n*, %)	22 (38.6)	26 (15.3)	**<0.001**
Episiotomy (*n*, %)	14 (24.6)	4 (2.4)	**<0.001**
OASI (*n*, %)	–	3 (1.8)	–
Vulvar tears (*n*, %)	1 (1.8)	9 (5.3)	0.3
Blood transfusion (*n*, %)	9 (15.8)	4 (2.4)	**0.02**

*Note*: Bold values denote statistical significance at the *p* < 0.05 level.

Abbreviations: BMI, body mass index; CD, cesarean delivery; IUGR, intrauterine growth restriction; GDM, gestational diabetes mellitus; PET, preeclampsia toxemia; PSS, prolonged second stage; OASI, obstetric anal sphincter injury.

In multivariable conditional logistic regression analysis (Table 2), several factors emerged as independent predictors of postpartum hematoma. Episiotomy (odds ratio [OR] 35.9, 95% confidence interval [CI] 5.6–232.4, *p* < 0.001), second‐degree laceration (OR 10.3, 95% CI 3.2–32.9, *p* < 0.001), and vacuum‐assisted delivery (OR 5.0, 95% CI 1.0–24.5, *p* = 0.047) were significantly associated with hematoma formation, while nulliparity demonstrated a non‐significant trend (OR 2.3, 95% CI 0.9–6.2, *p* = 0.094). Higher BMI was found to be protective (OR 0.69 per unit, 95% CI 0.55–0.85, *p* = 0.001), as was epidural analgesia (OR 0.33, 95% CI 0.12–0.93, *p* = 0.035).

**TABLE 2:** Multivariable conditional logistic regression analysis for predictors of postpartum hematoma.

Variable	aOR	95% CI	*p*‐value	Score^a^
BMI	0.685	0.551–0.853	**0.001**	−1^b^
Epidural	0.333	0.119–0.927	**0.035**	2^c^
Vacuum‐assisted delivery	5.004	1.022–24.512	**0.047**	2
Nulliparity	2.325	0.865–6.248	0.094	1
Second‐degree laceration	10.298	3.225–32.885	**<0.001**	2
Episiotomy	35.937	5.557–232.389	**<0.001**	3

*Note*: Bold values denote statistical significance at the *p* < 0.05 level.

Abbreviations: aOR, adjusted odds ratio; BMI, body mass index; CI, confidence interval.

^a^ The score was based on an unconditional multivariable logistic regression to increase generalizability.

^b^ For BMI > 25.

^c^ For not having epidural anesthesia.

**FIGURE 1:**
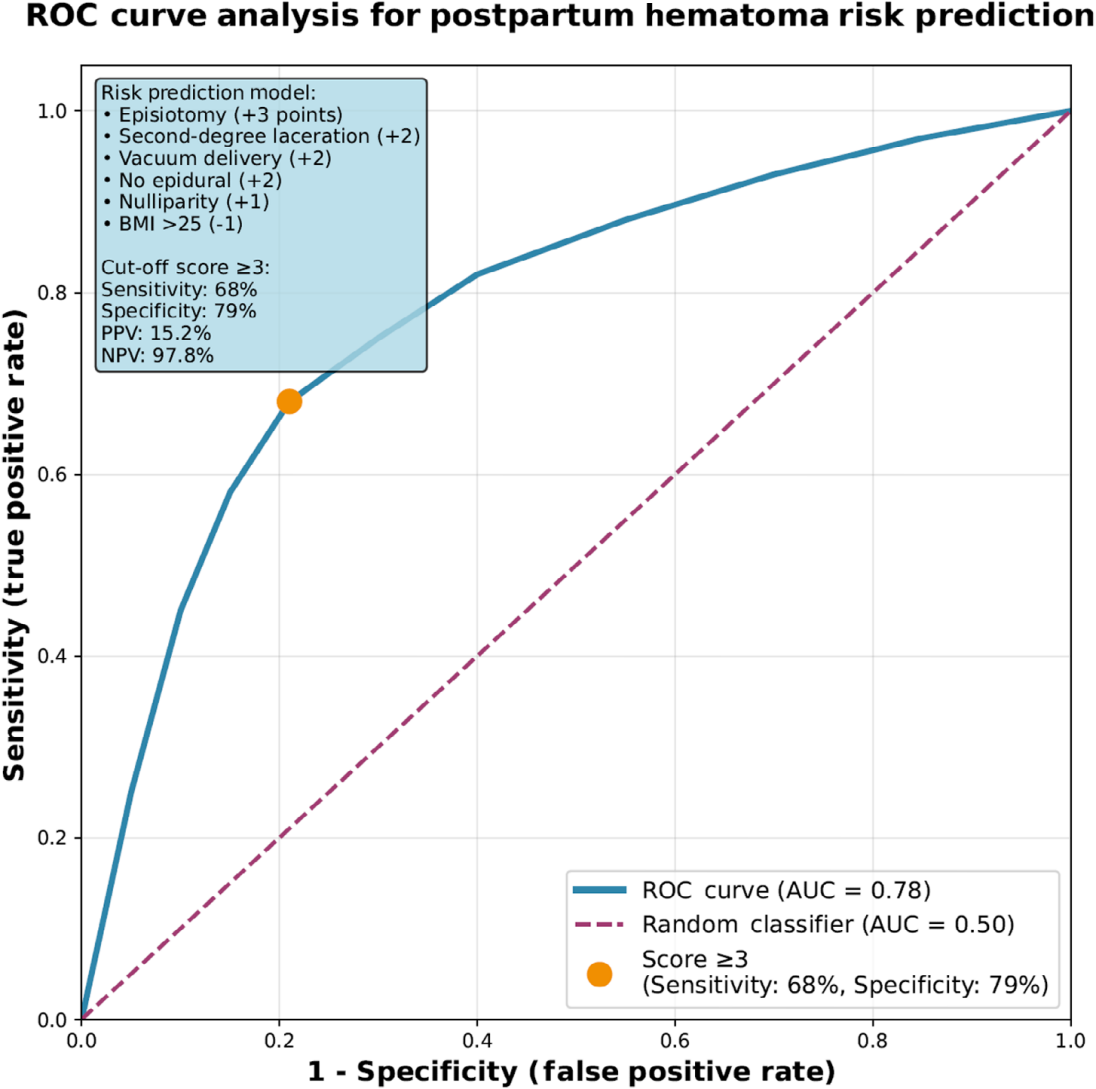
Receiver operating characteristic (ROC) curve analysis of a risk prediction score based on a multivariable model. AUC, area under the curve; ROC, receiver operating characteristic.

A clinical risk score was subsequently derived from the multivariable model, assigning weighted points according to the strength of association: episiotomy (3 points), second‐degree laceration (2 points), vacuum‐assisted delivery (2 points), absence of epidural analgesia (2 points), nulliparity (1 point), and BMI > 25 (−1 point). The model demonstrated good discriminative performance, with an AUC of 0.83 (95% CI 0.76–0.90). A score of ≥3 yielded a sensitivity of 68% and specificity of 79% for predicting hematoma occurrence (Figure 1). At this threshold, the PPV was 15.2% and the NPV was 97.8%, indicating that the model performs particularly well at identifying women at low risk in whom postpartum hematoma is unlikely.

Among the 57 women with hematomas, most (73.7%) were managed conservatively with observation and supportive care, while 15 (26.3%) required surgical intervention, performed at a mean of 6.5 ± 3.8 h after diagnosis (Table 3). The mean interval from delivery to diagnosis was 1.0 ± 1.6 days. A prolonged second stage of labor was the only factor significantly associated with surgical intervention (20.0% vs. 2.4%, *p* < 0.05). Non‐significant trends were observed for vacuum delivery (33.3% vs. 16.7%, *p* = 0.17), absence of epidural analgesia (60.0% vs. 42.9%, *p* = 0.25), and a greater mean hemoglobin decline (3.5 ± 1.0 vs. 2.9 ± 1.6 g/dL, *p* = 0.33).

**TABLE 3:** Subgroup analysis of women with postpartum hematoma: Comparison between those requiring surgical intervention and those who did not.

Characteristic	Surgical intervention, *N* = 15	No surgical intervention, *N* = 42	*p*‐value
Maternal age (mean, SD)	33.6 (4.01)	31.9 (4.6)	0.19
Gestational age (mean, SD)	39.03 (1.8)	39.02 (2.05)	0.98
Newborn weight (mean, SD)	3069 (429)	3195 (512)	0.39
BMI (mean, SD)	21 (1.3)	20.6 (5.7)	0.78
Previous CD (*n*, %)	1 (6.7)	3 (7.1)	0.95
Oxytocin (*n*, %)	6 (40.0)	21 (50.0)	0.51
PPH (*n*, %)	1 (6.7)	5 (11.9)	0.57
Epidural (*n*, %)	6 (40.0)	24 (57.1)	0.25
PSS (*n*, %)	2 (20.0)	1 (2.4)	**0.02**
Induction (*n*, %)	3 (20.0)	10 (23.8)	0.76
Vacuum (*n*, %)	5 (33.3)	7 (16.7)	0.17
Nulliparity (*n*, %)	9 (60.0)	32 (76.2)	0.23
First‐degree laceration (*n*, %)	2 (13.3)	8 (19.0)	0.62
Second‐degree laceration (*n*, %)	7 (46.7)	15 (35.7)	0.45
Episiotomy (*n*, %)	4 (26.7)	10 (23.8)	0.83
Vulvar tears (*n*, %)	–	1 (2.4)	–
Delta Hb (mean, SD)	3.5 (1.03)	2.9 (1.6)	0.33
Delta Hb > 3 g/dL (*n*, %)	7 (46.7%)	16 (38.1%)	0.56
Intrapartum fever (*n*, %)	–	2 (4.8)	0.40
Blood transfusion (*n*, %)	3 (20.0)	6 (14.3)	0.60

*Note*: Bold values denote statistical significance at the *p* < 0.05 level.

Abbreviations: BMI, body mass index; CD, cesarean delivery; PPH, postpartum hemorrhage; PSS, prolonged second stage; Hb, hemoglobin.

## 4 DISCUSSION

This matched case‐control study provides important insights into epidemiology, risk factors, and clinical management of postpartum hematomas.

Our main findings were as follows: (1) Postpartum hematoma is rare and occurred in 0.039% of all deliveries, corresponding to 0.05% of vaginal births. (2) Women who developed hematomas had lower BMI, were more often nulliparous, and had higher rates of episiotomy, second‐degree laceration, and vacuum‐assisted delivery. (3) In multivariable analysis, episiotomy, second‐degree laceration, and vacuum‐assisted delivery emerged as independent risk factors, while higher BMI and epidural analgesia were protective. (4) The derived clinical risk score demonstrated good discriminative ability (AUC 0.83), with a score ≥3 predicting hematoma occurrence with 68% sensitivity and 79% specificity. (5) Most hematomas were managed conservatively, while approximately one‐quarter required surgical intervention, primarily associated with a prolonged second stage of labor.

### 4.1 Our findings in the context of other observations

The incidence of postpartum hematoma is not routinely reported in large epidemiologic studies, but population‐based data indicate that vaginal hematoma is a rare complication of childbirth, occurring in approximately 0.1%–0.2% of deliveries [4, 10]. Our observed rate of 0.04% among vaginal births is somewhat lower yet remains within the range described in the literature. It is important to note that most of the available estimates are derived from cohorts managed in earlier practice eras, prior to the widespread adoption of standardized perineal protection techniques and more restrictive episiotomy policies [11]. Accordingly, our reduced incidence may reflect contemporary institutional practice patterns, particularly our restrictive approach to episiotomy and high rate of epidural utilization (∼70%), both of which are associated with decreased perineal trauma and are further elaborated in subsequent sections.

### 4.2 Risk factors

The identification of episiotomy as the strongest independent risk factor (OR 35.9) aligns with contemporary evidence demonstrating that, particularly for mediolateral episiotomies, it represents a significant risk factor [12, 13]. These findings underscore the need for restrictive episiotomy policies and support the historical observations of Morgans et al., who advocated for prophylactic measures following large episiotomy repairs [14]. Our results provide compelling evidence for careful consideration of episiotomy indications, reinforcing current recommendations from leading obstetric bodies that favor selective rather than routine episiotomy use [15, 16, 17].

The association between vacuum‐assisted delivery and hematoma formation (OR 5.0) is consistent with existing literature documenting increased perineal trauma risks with operative vaginal delivery. Large cohort and meta‐analytic data demonstrate maternal complication rates as high as 21% following instrumental delivery, with trauma rates ranging from 13% for vacuum to 25% for forceps, substantially higher than rates observed with spontaneous vaginal birth [18, 19, 20].

Second‐degree perineal lacerations emerged as another significant risk factor (OR 10.3), highlighting the importance of perineal protection techniques during delivery. This finding extends previous observations about the relationship between perineal trauma and hematoma formation, suggesting that even moderate perineal trauma may predispose to hematoma development [11].

These findings carry meaningful clinical implications. Births involving vacuum‐assisted delivery, episiotomy, or second‐degree perineal lacerations warrant increased vigilance and structured postpartum monitoring. Consistent with the management approach proposed by Tsumagari et al. [21], women with these risk factors should undergo early systematic perineal and vaginal assessment, close monitoring of vital signs and shock index, and scheduled re‐examination during the first postpartum hours to allow early detection of evolving swelling or pain.

Current literature emphasizes that higher birth weight is a significant risk factor for postpartum hematoma, particularly in the context of instrumental vaginal delivery or episiotomy. The medical literature consistently demonstrates that increased birth weight is associated with elevated rates of maternal trauma, including hematoma formation, due to greater tissue stretching and vascular injury during delivery [4]. In contrast to previous reports, in our cohort birth weight did not differ significantly between cases and controls in univariate analysis. This may be explained by the limited number of hematoma cases and, consequently, insufficient statistical power to detect modest associations.

### 4.3 Protective factors

Traditional epidural analgesia is associated with a higher rate of instrumental delivery and prolonged second stage of labor [22], both of which are established risk factors for perineal trauma. However, direct evidence linking epidural use to increased rates of vaginal or vulvar hematoma is lacking [23, 24, 25]. In contrast, our study identified a protective effect of epidural analgesia (OR 0.33), representing a novel finding with potential clinical relevance. This association may be explained by several mechanisms, including improved maternal cooperation during delivery and during perineal repair, reduced perineal muscle tension, and more controlled delivery techniques when women are comfortable [26]. These findings challenge traditional concerns regarding epidural use and suggest potential benefits beyond analgesia.

The protective effect of higher BMI (OR = 0.69) is intriguing and may relate to tissue characteristics, vascular anatomy, or other physiological factors. While obesity is associated with numerous obstetric complications, our findings suggest that low BMI may predispose to hematoma formation, possibly through differences in tissue resilience or vascular fragility [27, 28]. This observation is consistent with current literature: several large cohort and retrospective studies have demonstrated that higher maternal BMI is associated with a decreased risk of minor perineal trauma and obstetric anal sphincter injury during vaginal delivery, with the odds of severe tears decreasing as BMI increases [29, 30]. Durnea et al. reported that the risk of minor perineal trauma was significantly reduced among women with obesity (OR = 0.91) and those with severe obesity (OR = 0.87) compared with women of normal BMI [30].

### 4.4 Clinical risk stratification and surgical management considerations

The pathophysiology of postpartum hematomas involves bleeding from damaged vessels in the birth canal that becomes contained within tissue planes, creating expanding masses that may not be immediately apparent [31]. Unlike overt postpartum hemorrhage, hematomas can develop insidiously and may present hours to days after delivery, making early recognition challenging. Therefore, our risk prediction model (AUC 0.83), which includes episiotomy, second‐degree laceration, vacuum‐assisted delivery, epidural analgesia, nulliparity, and BMI > 25, provides a practical tool for clinical risk stratification that could complement existing postpartum monitoring protocols. The identification of high‐risk patients (score ≥3) could inform targeted monitoring strategies, including more frequent postpartum assessments, enhanced nursing vigilance, and consideration of imaging when symptoms develop. In women who are clinically stable, early diagnosis may allow for conservative management with close monitoring in the labor ward, administration of tranexamic acid, temporary delay of venous thromboembolism prophylaxis when appropriate, and extended recovery room observation to ensure hemodynamic stability, while reserving surgical evacuation with dead‐space closure or selective transcatheter arterial embolization for cases with ongoing expansion or clinical deterioration [10, 32, 33]. Notably, the model's high negative predictive value (97.8%) suggests that a low‐risk score reliably excludes postpartum hematoma among women presenting with nonspecific perineal pain or swelling, potentially reducing unnecessary imaging or interventions and helping remove hematoma from the differential diagnosis when clinical findings are equivocal.

In our study, 26.3% of women with postpartum hematomas required surgical intervention, with a mean interval of 6.5 h from delivery to treatment, highlighting the acute nature and potential severity of this complication. Although direct evidence linking prolonged second stage to surgical management of hematomas is limited, prolonged second stage is consistently associated with increased obstetric soft‐tissue trauma [34, 35]. In our cohort, prolonged second stage was associated with the need for surgical intervention in univariate analysis. This likely reflects greater tissue disruption and more extensive vascular injury, resulting in larger or deeper hematomas that are less amenable to conservative management. The sustained compression and distension of the pelvic soft tissues during prolonged pushing may lead to impaired natural capacity for tamponade and therefore ongoing or expanding bleeding [35, 36].

### 4.5 Clinical implications

This study identifies clear risk factors for postpartum vaginal and vulvar hematomas, while highlighting the protective roles of epidural analgesia and higher BMI. These findings support the need for heightened postpartum surveillance in women with these risk factors, including early perineal assessment, structured monitoring of symptoms and vital signs, and consideration of early imaging when pain or swelling is reported. The risk score developed in this study, which demonstrated a high NPV, may help clinicians reliably identify women at low likelihood of hematoma, potentially reducing unnecessary evaluations. Integration of the risk score into routine practice still requires external validation before it can be confidently adopted in clinical decision‐making.

### 4.6 Research implications

Several important questions remain unanswered. Future prospective studies should validate the identified risk factors and the proposed risk score across diverse populations and clinical settings to confirm their generalizability. Additional research is needed to refine early diagnostic pathways, evaluate optimal monitoring strategies for women at risk, and determine whether targeted interventions, such as prophylactic postpartum tranexamic acid for high‐risk women, can reduce hematoma progression or the need for surgery. Larger multicenter cohorts may also help clarify predictors of surgical intervention among women who develop hematomas.

### 4.7 Strengths and limitations

Our study has several strengths. First, to our knowledge, this is one of the largest matched case–control analyses of postpartum vaginal and vulvar hematomas, drawn from a cohort of over 113,000 vaginal deliveries. Second, the matched design (by maternal and gestational age) and the detailed manual extraction of clinical data allowed for accurate characterization of risk factors, minimizing selection and misclassification bias. Third, the use of multivariable conditional logistic regression enabled identification of independent predictors, from which we derived a clinically applicable risk score with good discriminative performance. Finally, we examined predictors of surgical intervention, an aspect that has received limited attention in previous studies and has direct relevance for bedside decision‐making and resource allocation. However, this study also has limitations. Its retrospective design introduces the possibility of documentation and selection bias, particularly as smaller or asymptomatic hematomas may have been underdiagnosed. Although clinical examination was the primary diagnostic modality, imaging was not performed uniformly, which may lead to under‐recognition of deeper paravaginal hematomas. In addition, it remains unclear whether hematomas were suspected due to localized symptoms (e.g., pain or pressure) or systemic signs (e.g., symptomatic anemia or a significant hemoglobin drop). Furthermore, the rarity of the condition resulted in a relatively small number of cases despite the large source population, which may limit the power to detect more subtle associations and preclude multivariable modeling for surgical intervention. This rarity, combined with the infrequent occurrence of certain risk factors among controls and potential multicollinearity between interventions like vacuum and episiotomy, led to increased standard errors and wide confidence intervals. Consequently, larger multicenter studies are required to provide more precise effect sizes and enhance the statistical power of these associations. Additionally, the risk score was developed and tested within the same population and therefore requires external validation before clinical implementation. Finally, the study was conducted at a single, university‐affiliated tertiary medical center, and the results may not be generalized to other settings.

## 5 CONCLUSION

Postpartum hematomas, while uncommon, represent a significant clinical challenge with predictable risk factors and management requirements. Episiotomy, vacuum delivery, and second‐degree lacerations substantially increased risk, while epidural analgesia and higher BMI appear protective. Early recognition through risk stratification, combined with appropriate use of imaging and interventional techniques, may improve outcomes and reduce the need for surgical intervention.

## AUTHOR CONTRIBUTIONS

Itai Atar and Emmanuel Attali conceived and designed the study. Itai Atar, Einat Tako, Daniel Gabbai, Itamar Gilboa, and Yariv Yogev contributed to data acquisition and chart review. Itai Atar performed the statistical analysis with supervision from Emmanuel Attali. Itai Atar drafted the manuscript. Emmanuel Attali, Yariv Yogev, and Itamar Gilboa provided critical revisions for important intellectual content. All authors reviewed and approved the final manuscript.

## CONFLICT OF INTEREST STATEMENT

The authors declare no conflicts of interest.

## FUNDING INFORMATION

The authors received no specific funding for this work.

## Data Availability

The data that support the findings of this study are derived from electronic medical records and contain personally identifiable health information. Due to institutional and ethical restrictions, these data cannot be publicly shared. De‐identified data may be made available from the corresponding author upon reasonable request.

## References

[1] Ridgway, L. E. 1995. “Puerperal Emergency. Vaginal and Vulvar Hematomas.” Obstetrics and Gynecology Clinics of North America 22(2): 275–82. 7651671

[2] Kawashima, M. and H. Tokushige . 2021. “Analysis of Puerperal Hematoma: A Retrospective Study.” Journal of Rural Medicine 16(3): 2020–46. 10.2185/jrm.2020-046. PMC8249368 34239624

[3] Dorland, T. R. , H. F. Rabarikoto , J. G. Raelison , D. M. A. Randriambololona , T. A. Rajaonera , H. Andrianampanalinarivo . 2014. “Hematoma Vulvo‐Vaginal: Exceptional Etiology of Obstetrical Near Miss.” Pan African Medical Journal 19: 167.

[4] Saleem, Z. and H. Rydhström . 2004. “Vaginal Hematoma During Parturition: A Population‐Based Study.” Acta Obstetricia Et Gynecologica Scandinavica 83(6): 560–2. 10.1111/j.1600-0412.2004.00535.x. 15144338

[5] Bihan, L. , E. Nowak , F. Anouilh , C. Tremouilhac , P. Merviel , C. Tromeur , S. Robin , et al. 2022. “Development and Validation of a Predictive Tool for Postpartum Hemorrhage After Vaginal Delivery: A Prospective Cohort Study.” Biology 12(1): 54. 10.3390/biology12010054. PMC9855728 36671746

[6] Committee on Practice Bulletins‐Obstetrics . 2017. “Practice Bulletin No. 183: Postpartum Hemorrhage.” Obstetrics and Gynecology 130(4): e168–e86. 10.1097/AOG.0000000000002351. 28937571

[7] Distefano, M. , L. Casarella , S. Amoroso , C. Di Stasi , G. Scambia , and G. Tropeano . 2013. “Selective Arterial Embolization as a First‐line Treatment for Postpartum Hematomas.” Obstetrics and Gynecology 121(2 Pt 2 Suppl 1): 443–7. 10.1097/aog.0b013e31827d90e1. 23344403

[8] Hiersch, L. , R. Bergel‐Bson , D. Asher , A. Aviram , R. Gabby‐Benziv , Y. Yogev , E. Ashwal . 2017. “Risk Factors for Post‐partum Hemorrhage Following Vacuum Assisted Vaginal Delivery.” Archives of Gynecology and Obstetrics 295(1): 75–80. 10.1007/s00404-016-4208-5. 27683268

[9] Chawanpaiboon, S. , V. Titapant , and J. Pooliam . 2023. “Maternal Complications and Risk Factors Associated With Assisted Vaginal Delivery.” BMC Pregnancy Childbirth 23(1): 756. 10.1186/s12884-023-06080-9. PMC10601252 37884886

[10] Zahn, C. M. , G. D. Hankins , and E. R. Yeomans . 1996. “Vulvovaginal Hematomas Complicating Delivery. Rationale for Drainage of the Hematoma Cavity.” Journal of Reproductive Medicine 41(8): 569–74. 8866383

[11] Okeahialam, N. A. , A. H. Sultan , and R. Thakar . 2024. “The Prevention of Perineal Trauma During Vaginal Birth.” American Journal of Obstetrics and Gynecology 230(S3): S991–S1004. 10.1016/j.ajog.2022.06.021. 37635056

[12] İskender, C. , H. O. Topçu , H. Timur , A. Oskovi , G. Göksu , A. Sucak , N. Danışman . 2016. “Evaluation of Risk Factors in Women With Puerperal Genital Hematomas.” The Journal of Maternal‐Fetal & Neonatal Medicine 29(9): 1435–S9. 10.3109/14767058.2015.1051018. 26043648

[13] Sagi‐Dain, L. and S. Sagi . 2015. “Morbidity Associated With Episiotomy in Vacuum Delivery: A Systematic Review and Meta‐Analysis.” BJOG 122(8): 1073–81. 10.1111/1471-0528.13439. 25950083

[14] Morgans, D. , N. Chan , and C. A. Clark . 1999. “Vulval Perineal Haematomas in the Immediate Postpartum Period and Their Management.” Australian and New Zealand Journal of Obstetrics and Gynaecology 39(2): 223–7. 10.1111/j.1479-828x.1999.tb03378.x. 10755785

[15] Houlbracq, R. , C. Le Ray , B. Blondel , N. Lelong , A. A. Chantry , and T. Desplanches . 2025. “Episiotomies and Obstetric Anal Sphincter Injuries following a Restrictive Episiotomy Policy in France: An Analysis of the 2010, 2016, and 2021 National Perinatal Surveys.” PLoS Medicine 22(1): e1004501. 10.1371/journal.pmed.1004501. PMC11731868 39808593

[16] Sagi‐Dain, L. , I. Kreinin‐Bleicher , R. Bahous , N. Gur Arye , T. Shema , A. Eshel , O. Caspin , et al. 2020. “Is it Time to Abandon Episiotomy Use? A Randomized Controlled Trial (EPITRIAL).” International Urogynecology Journal 31(11): 2377–85. 10.1007/s00192-020-04332-2. 32448935

[17] Jiang, H. , X. Qian , G. Carroli , and P. Garner . 2017. “Selective versus Routine Use of Episiotomy for Vaginal Birth.” Cochrane Database of Systematic Reviews 2(2): CD000081. 10.1002/14651858.CD000081.pub3. PMC5449575 28176333

[18] Bienstock, J. L. , A. C. Eke , and N. A. Hueppchen . 2021. “Postpartum Hemorrhage.” New England Journal of Medicine 384(17): 1635–45. 10.1056/NEJMra1513247. PMC10181876 33913640

[19] Murphy, D. J. , R. E. Liebling , L. Verity , R. Swingler , and R. Patel . 2001. “Early Maternal and Neonatal Morbidity Associated With Operative Delivery in Second Stage of Labour: A Cohort Study.” Lancet 358(9289): 1203–7. 10.1016/S0140-6736(01)06341-3. 11675055

[20] Abdi, H. B. , E. B. Wakwaya , B. S. Damtew , B. A. Hussen , and T. K. Beyen . 2025. “Maternal Complication of Instrumental Vaginal Delivery and Its Associated Factors: Systematic Review and Meta‐Analysis.” PLoS ONE 20(4): e0320003. 10.1371/journal.pone.0320003. PMC12021129 40273174

[21] Tsumagari, A. , R. Ohara , M. Mayumi , H. Yagi , Y. Nagai , M. Obata‐Yasuoka , H. Hamada , et al. 2019. “Clinical Characteristics, Treatment Indications and Treatment Algorithm for Post‐partum Hematomas.” Journal of Obstetrics and Gynaecology Research 45(6): 1127–33. 10.1111/jog.13943. 30788889

[22] Eltzschig, H. K. , E. S. Lieberman , and W. R. Camann . 2003. “Regional Anesthesia and Analgesia for Labor and Delivery.” New England Journal of Medicine 348(4): 319–32. 10.1056/NEJMra021276. 12540646

[23] Dominsky, O. , E. Attali , U. Amikam , R. Gold , C. Greenberger , Y. Yogev , Y. Baruch . 2025. “The Association Between Epidural Analgesia and Perineal Injury in Primiparous Women: A Propensity Score‐Matched Cohort Study.” International Journal of Gynaecology and Obstetrics 173(1):144–50. 10.1002/ijgo.70578. PMC12988395 41048114

[24] Rosero, E. B. and G. P. Joshi . 2016. “Nationwide Incidence of Serious Complications of Epidural Analgesia in the United States.” Acta Anaesthesiologica Scandinavica 60(6): 810–20. 10.1111/aas.12702. 26876878

[25] Sachs, M. K. , E. Kapfhammer , R. Brun , L. Kandler , N. Ochsenbein , and C. Haslinger . 2025. “Epidural Anesthesia During Labor and Delivery and Postpartum Hemorrhage.” Journal of Perinatal Medicine 53(7): 871–6. 10.1515/jpm-2024-0567. 40418836

[26] Genç Koyucu, R. , F. Ketenci Gencer , S. R. Bilici . 2025. “Effects of Manual Perineal Protection and Pushing Techniques Used in the Second Stage of Labor on Perineal Outcomes: A Randomized Controlled Trial of Combinations of Strategies.” BMC Pregnancy Childbirth. 25(1): 671. 10.1186/s12884-025-07564-6. PMC12180208 40537769

[27] Roy, P. , M. S. Sujatha , B. Biswas , A. Chatterjee , P. Roy . 2015. “A Comparative Study of Perineal Morbidity in Vaginal Delivery With and Without Episiotomy.” International Journal of Reproduction, Contraception, Obstetrics and Gynecology 4(6): 1727–34.

[28] Chamsi, A. T. 2018. “Perineal Tears Incidence and Risk Factors; A Four Years Experience in a Single Saudi Center.” Interventions in Gynaecology and Women's Healthcare 1(5): 122–8. 10.32474/IGWHC.2018.01.000122.

[29] Shalabna, E. , M. Yinon , Y. Daykan , W. Assaf , Y. Abramov , and L. Sagi‐Dain . 2024. “The Association Between BMI, Episiotomy, and Obstetric Anal Sphincter Injuries (OASIS) in Singleton Vaginal Deliveries.” European Journal of Obstetrics, Gynecology, and Reproductive Biology 299: 143–7. 10.1016/j.ejogrb.2024.06.011. 38865741

[30] Durnea, C. M. , A. E. Jaffery , N. Gauthaman , and S. K. Doumouchtsis . 2018. “Effect of Body Mass Index on the Incidence of Perineal Trauma.” International Journal of Gynaecology and Obstetrics 141(2): 166–70. 10.1002/ijgo.12403. 29178349

[31] Girault, A. , C. Deneux‐Tharaux , L. Sentilhes , F. Maillard , and F. Goffinet . 2018. “Undiagnosed Abnormal Postpartum Blood Loss: Incidence and Risk Factors.” PLoS ONE 13(1): e0190845. 10.1371/journal.pone.0190845. PMC5761868 29320553

[32] Takahashi, T. , H. Tomita , H. Hamada , M. Tadakawa , N. Iwama , and M. Saito . 2024. “Transcatheter Arterial Embolization Outperforms Surgery in Reducing Blood Transfusions for Postpartum Vulvovaginal Hematoma.” American Journal of Obstetrics and Gynecology 231(6): 653.e1–e8. 10.1016/j.ajog.2024.03.016. 38518850

[33] Gutierrez, A. J. , K. Dawodu , K. H. Mayer , and A. L. McGill . 2022. “Treatment Strategies for Obstetric Puerperal Genital Hematomas: A Case Series.” Obstetrics and Gynecology 140(3): 383–6. 10.1097/AOG.0000000000004871. 35926214

[34] Stephansson, O. , A. Sandström , G. Petersson , A. K. Wikström , and S. Cnattingius . 2016. “Prolonged Second Stage of Labour, Maternal Infectious Disease, Urinary Retention and Other Complications in the Early Postpartum Period.” BJOG 123(4): 608–16. 10.1111/1471-0528.13287. PMC6680275 25601143

[35] Young, C. , S. Bhattacharya , A. Woolner , A. Ingram , N. Smith , E. A. Raja , M. Black . 2023. “Maternal and Perinatal Outcomes of Prolonged Second Stage of Labour: A Historical Cohort Study of Over 51,000 Women.” BMC Pregnancy Childbirth 23(1): 467. 10.1186/s12884-023-05733-z. PMC10288707 37349683

[36] ACOG Publication . 2024. “First and Second Stage Labor Management ACOG Clinical Practice Guideline No. 8.” Obstetrics & Gynecology 143(1):144–62. 10.1097/AOG.0000000000005447. 38096556

